# In favor of behavior: on the importance of experimental paradigms in testing predictions from Gray's revised reinforcement sensitivity theory

**DOI:** 10.3389/fnsys.2014.00184

**Published:** 2014-09-30

**Authors:** Sebastian Markett, Christian Montag, Martin Reuter

**Affiliations:** ^1^Department of Psychology, University of BonnBonn, Germany; ^2^Center for Economics and Neuroscience, University of BonnBonn, Germany; ^3^Department of Psychology, University of UlmUlm, Germany

**Keywords:** anxiety, fear, approach/avoidance, affective neuroscience, reinforcement sensitivity theory (RST)

Approach to desirable events or stimuli of reward and avoidance of undesirable events or stimuli of non-reward or punishment are powerful forces to drive behavior. The importance of approach and avoidance motivation has been outlined in many psychological theories and traditions, including psychodynamics, behaviorism and cognitivism (Elliot and Covington, 2001). Of all theoretical accounts, the *Reinforcement Sensitivity Theory* (RST) by Jeffrey Gray is probably the most elaborate perspective on approach and avoidance (Gray, 1971; Gray and McNaughton, 2000). Key contributions by Gray within this framework are the identification of neural circuits underlying approach and avoidance behavior, the description of these circuits as neuropsychological systems with circumscribed functions in terms of a *conceptual nervous system*, and the development of a theory of personality based on individual differences in the reactivity of these systems.

The plethora of empirical findings in response to the initial formulation of RST has led to a major revision of the theory (Gray and McNaughton, 2000). In the present commentary we focus on one key aspect of the revised RST, namely the processing of (non-)ambiguous dangerous stimuli, which plays a crucial role in disentangling the emotions of fear and anxiety. The revised RST distinguishes between the behavioral inhibition system (BIS) mediating anxiety and the fight flight freezing system (FFFS) reflecting fear. Whereas anxiety represents the emotion elicited when approaching potential threat (for example in foraging) fear represents a “get me out of here” emotion that operates during active avoidance of non-ambiguous threat and governs flight behavior or alternatively freezing or fight if flight is not an option (for example in the immediate vicinity of a predator). Both emotions are conceptualized as distinct entities depending on defensive direction with careful approach behavior in the context of potentially dangerous stimuli to clarify the stimuli's nature vs. avoidance of a clear threat. The BIS and FFFS are implemented in distinct but parallel neural streams (McNaugthon and Corr, 2006): Both neural streams are hierarchically organized along a rostral-cortical (e.g., cingulate, prefrontal cortex) to caudal-subcortical (e.g., periaqueductal gray, hypothalamus, amygdala) axis. Within each structure, different nuclei or subdivisions activate either fear or anxiety. Defensive distance, which is the perceived intensity of threat (Blanchard and Blanchard, 1990), determines which level of the hierarchy with its associated behavioral output becomes active in a given situation. Rostral regions along the hierarchy are thought to react to more distal threat while the caudal regions are activated by proximal threat (especially the periaqueductal gray). The functioning of the BIS (i.e., the emotion of anxiety), however, is not restricted to the conflict that emerges in the tension between approach to, vs. avoidance of potential threat but generalizes to all forms of conflict that results from incompatible goals such as avoidance/avoidance and approach/approach conflict.

The refinements to RST have been based on experimental work in rodents but the theory claims validity for human affective processing and personality as well. In animal research the emotion of anxiety has been investigated with a so called “Visible Burrow System” (e.g., Blanchard and Blanchard, 1989), where a rat is placed in a setting prepared with cat odor in the absence of an actual cat (Blanchard et al., 2001). To a rat, a cat is a clear threat but since its precise location cannot be inferred from its odor alone, flight is not likely to lead to safety. The different information processed by the visual and olfactory senses results in a conflict that triggers the behavioral inhibition system. By the activation of the BIS the rat stops its present exploration behavior (searching for food or a mate) and orients itself toward the potential danger. The rat resolves the conflict by obtaining further information through careful approach behavior. In consequence, either the behavioral approach system (BAS) is activated to return to the exploration of the environment or the FFFS to cope with the immediate danger (the latter is not the case in the Visible Burrow system paradigm).

The aforementioned cross species translation from rodents to humans requires empirical validation. For the design of validation studies, it is important to keep in mind that RST is composed of two main components: A state component that describes neural systems including their reactivity and their associated behavioral and emotional output and a trait component describing stable behavioral dispositions arising from individual differences in the neural systems' sensitivity (Corr and McNaughton, 2006). Initially, RST set out in an Eysenckian tradition as a biologically oriented theory on human individual differences at the trait level (Corr and Perkins, 2006) but the refinement of the hypothesized neurobehavioral systems (the BIS, the FFFS, and the BAS) in the theory's revision have been derived on the basis of ethopharmacological experiments and lesion studies in rodents. Importantly, the animals used in these studies were unselected rats, i.e., no different strains of rats bred for individual differences in fear- or anxiety proneness were used. Thus, the initial formulation of the revised RST has been based on the analysis of states and changes in states after experimental manipulation rather than traits. This highlights the fact that RST is not only a theory on personality but a more general theory on emotion, motivation and learning (Smillie et al., 2006).

So far, the majority of translational work has focused on human individual differences on the trait level, either by assessing predicted relationships between trait anxiety and trait fear (e.g., Perkins et al., 2007; Smederevac et al., 2014) or by testing the influence of individual differences in relevant traits on behavioral states (for meta-analysis see Leue and Beauducel, 2008). While these literatures were successful in testing important predictions within the scope of RST, they come with the downside that the use of self-report measures alone leaves a large body of RST untouched. Particularly, they do not allow for inference on the neural and neurochemical implementation of the hypothesized systems if no biological data is added to these studies. Furthermore, any attempt to link personality traits to individual differences in behavior or the reactivity of neural systems requires structurally valid psychometric tools. Special caution must be taken upon selecting such personality questionnaires in order to avoid circularity: As a structurally valid personality questionnaire is ideally constructed on the basis of the theory under scrutiny, any favorable result can be interpreted as evidence for the measurement tool or for the theory's prediction.

Here we argue for the importance of well-designed experimental paradigms that are crucial in the endeavor of translating non-human animal findings to our species. We hold that it is particularly important to derive paradigms that focus on main effects of systematically varied experimental context and pharmacological manipulation on recordable behavior and brain activity. The study of transient states rather than stable traits is an important step toward establishing general causal systems of human behavior that can be subjected to the study of individual differences in a second step. Of course, the problem of circularity mentioned above is not restricted to self-report measures and can also apply to behavioral tasks. To avoid or to reduce this problem, behavioral tasks need to be designed to resemble the animal tasks on which the refinements of RST are based. If such a translated task responds to different classes of drugs in humans as predicted from animal data, it can be confidentially used to test the individual differences part of the theory as well.

Important questions in the context of revised RST are: Can fear and anxiety be dissociated in humans? Are fear and anxiety elicited by active avoidance of and approach to threat, respectively? Can fear and anxiety be disentangled by biological markers like for example gene polymorphisms? And, are fear and anxiety modulated by panicolytic and anxiolytic drugs similar to pharmacological effects in rodents? Perkins and colleagues have addressed some of these questions with an experimental task that has been derived from a typical behavioral protocol in rodents (Perkins et al., 2009). In the joystick-operated runway task (JORT), participants determine an onscreen dot's speed in a runway by controlling a joystick. The dot is chased by another onscreen dot and if caught, a highly unpleasant burst of white noise is emitted to the participant via headphones. Thus, participants are motivated to escape the chasing dot and the amount of force participants exert on the joystick can be interpreted as a proxy for active avoidance or in other words fear. In a second version of the task, the participant's dot is not only chased by one dot, there is also another dot in the runway running in front of the participant's dot. The unpleasant burst of noise is not only emitted when the participant's dot gets caught but also if it is run into the dot upfront. Thus, in order to escape the chasing dot, participants have to approach a potential threat, which is experienced as conflict or, in other words, anxiety. The emotion of anxiety is quantified by the amount of back- and forth oscillations with the joystick handle while avoiding both threatening dots. Key findings from pharmacological experiments with the JORT are that anxiolytic but not anti-panic drugs affect anxiety in terms of approach-withdrawal oscillations (Perkins et al., 2009). A similar relationship between anti-panic drugs and active avoidance, however, was not observed. Active avoidance in the JORT was however affected by a risk gene variant for panic disorder as predicted by RST (Perkins et al., 2011). The study of genetic variation is also a feasible means to probe neurotransmitter systems and has the advantage of being free from side effects. In contrast to RST predictions, however, a more recent study confirmed a dose dependent effect of an anxiolytic drug on active avoidance, an effect that depended on baseline individual differences on a fear scale (Perkins et al., 2013). Taken together, the JORT was successful in confirming three out of five predictions. It failed, however, to provide evidence for the core theme of the revised RST, a double dissociation of fear and anxiety, either on the pharmacological or on the molecular genetic level. This emphasizes the requirement to validate the state part of the theory separately from the trait part. Replication of these results, however, is needed to justify refinement of human RST, especially since other behavioral experiments have confirmed that fear and anxiety can be separated in human facial expressions (Perkins et al., 2012).

A general problem with translational fear research is that levels of experimentally induced fear might vary considerably across species and only a rather small amount of fear may be triggered in humans for ethical reasons. To parallel findings from rodent studies, it might be wise to use innate (but harmless) fear stimuli such as spiders, which are likely to elicit phobic reactions in humans. Mobbs et al. (2010) varied the proximity of a huge tarantula to the feet of healthy human participants while they were undergoing neuroimaging to test the effect of threat proximity on the responsiveness of different levels of the neural fear hierarchy. This paradigm activates the FFFS because a dangerous stimulus is presented that is unavoidable. As predicted, distant threats were more likely to activate the rostral part of the fear axis (such as prefrontal cortices) while proximal threats activated the caudal parts of the hierarchy such as the amygdala or the periaqueductal gray. Similar findings were obtained by the same group (Mobbs et al., 2007, 2009) using a pacman-style computer game where the participant was chased through a labyrinth and was subjected to a painful cutaneous electric shock when caught. While these experimental protocols were successful in testing the neural fear hierarchy and its dependence upon defensive distance, they do not allow any conclusion on the anxiety hierarchy or on the dissociation of fear and anxiety. The combination of ethopharmacology with neuroimaging or etho-genetic imaging studies (e.g., Montag et al., 2008) will help to shed light on these questions.

Behavioral experiments as described above will overcome issues of social desirability in answering self-reports and provide ecologically valid data on human behavior—if operationalized in the correct way. An automated fear response elicited by an approaching spider, and the threat of loud bursts or electric shocks will be more instructive for neuropsychological theories than a questionnaire score that results from the reflection on past behavior, behavioral dispositions, and emotional states.

## Conflict of interest statement

The authors declare that the research was conducted in the absence of any commercial or financial relationships that could be construed as a potential conflict of interest.
